# Improvement of tardive dyskinesia in a depressive patient treated with fluvoxamine

**DOI:** 10.1192/j.eurpsy.2023.437

**Published:** 2023-07-19

**Authors:** G. Dzhupanov

**Affiliations:** State Psychiatric Hospital for treatment of drug addiction and alcoholism, Sofia, Bulgaria

## Abstract

**Introduction:**

Depressive patients often receive antipsyhotics as ad-on treatmnent due to different reasons. Rare side effect, but with high potential for chronicity is tadrive dyskinesia. Standart treatment of this incapacitating condition includes tetrabenasine, valbenasine (not available in Bulgaria), tiapride, and strategies with adding antipsychotics. In case of lack of medication or therapeutic failure we face therapeutic dilemma. Fluvoxamine, an SSRI and σ1-receptor agonist, has been shown in case studies to be beneficial, and this confirmed in this case.

**Objectives:**

Description of improvement of tardive dyskinesia in a patient suffering from depression after switching antidepressive therapy to fluvoxamine.

**Methods:**

Study of a case of switching to fluvoxamine, based on review of relevant literature and own previous experience of treating other hypekinetic disorders – tics, with the same medicine.

**Results:**

A fifty-nine year old female patient suffering from long term depression received different antidepressants but also different mood stabilizers, anxiolitics and antipsyhotics (typical and atypical) as add-on treatment due to resistance, severe insomnia and anxiety, including even clozapine. Combination of paroxetine and clozapine resulted in improvement of sleep anxiety and tension, but with marked sedation as a side effect. Medications were successfully tapered off and replaced with trazodone and pregabaline. Soon however oral dyskinesia occurred. Patient developed hypersensitivity reaction when treated with tiapride. After switching antidepressant to fluvoxamine dyskinesia improved substantially.T

**Conclusions:**

This case demonstrates the potential of fluvoxamine in treatment of tardive dyskinesia. This effect is most probably result of σ1-receptor agonism of fluvoxamine.

**Disclosure of Interest:**

None Declared

